# Foliar Application of Potassium Salts to Olive, with Focus on Accompanying Anions

**DOI:** 10.3390/plants12030472

**Published:** 2023-01-19

**Authors:** Héctor A. Bahamonde, Carlos Pimentel, Luis Adrián Lara, Vikingur Bahamonde-Fernández, Victoria Fernández

**Affiliations:** 1 Facultad de Ciencias Agrarias y Forestales, Universidad Nacional de La Plata, Diagonal 113 N_ 469, La Plata 1900, Argentina; 2 Université Grenoble Alpes, Université Savoie Mont Blanc, CNRS, IRD, Université Gustave Eiffel, ISTerre, 38000 Grenoble, France; 3 Systems and Natural Resources Department, School of Forest Engineering, Universidad Politécnica de Madrid, 28040 Madrid, Spain; 4 Natural Resources Institute, Universidad Nacional de la Patagonia Austral, Lisandro de la Torre 1070, Río Gallegos 9400, Argentina

**Keywords:** epidermis, counterions, foliar fertilization, leaf uptake, mineral nutrition, nutrient salts, point of deliquescence, point of efflorescence

## Abstract

Potassium (K) is an essential element, which is often supplied to horticultural crops via foliar spraying. Some studies have investigated the effect of different foliar-applied K compounds; however, most studies have focussed on crop quality and yield parameters, or were performed with isolated leaf cuticles. The aim of this study was to evaluate the rates of the foliar ion penetration and leaf surface deposition of 130 mM K sprays of compounds with markedly different point of deliquescence (POD) and efflorescence (POE) values, the rates having been previously estimated in climate chamber trials. Shoots of field-grown, commercial olive trees were sprayed with K-nitrate (KNO_3_), K-sulphate (K_2_SO_4_), K-chloride (KCl), K-phosphate (K_3_PO_4_), K-carbonate (K_2_CO_3_) and K-bicarbonate (KHCO_3_), and leaf samples were collected after 3 and 24 h. Cation and anion concentrations were determined in the leaf tissues, and in a preliminary leaf water wash for estimating surface-deposited ion concentrations. No significant leaf tissue K increments were recorded between the K sprays. Olive tissue anion concentrations showed different patterns, and a chloride (Cl^−^) increase was detected 3 h after the foliar KCl supply. On the other hand, the foliar K applications led to leaf nitrate changes regardless of the K source supplied. High amounts of K and accompanying ions were recovered in the washing liquid of the foliar K-supplied leaves. Some foliar K treatments increased the leaf surface concentration of sulphate and chloride, suggesting a potential effect on leaf cell anion extrusion. Hence, despite no evidence of foliar K uptake, an effect of leaf anion concentrations was observed, indicating that foliar nutrient sprays may influence leaf and leaf surface anion balance.

## 1. Introduction

Potassium (K) is an essential element for plant growth, being necessary for enzyme activity, osmotic regulation and stomatal movement, among other functions [1]. This macronutrient is also important for improving crop production under biotic and abiotic stress factors [2]. On the other hand, soil K availability in many areas of the world is often low, and insufficient to cover crop K requirements: for this reason, K fertilisation is a common agricultural practice for ensuring crop yield and quality, and for improving plant tolerance of stress factors [1].

Olive (*Olea europaea*) is one of the most important crops in the Mediterranean area, where it has been cultivated for thousands of years [3,4]. In Spain, olive plantations occupy a surface area of 2.8 million hectares. Spain is the largest world exporter of olive oil [5]. Olive trees are adapted to grow in drylands under the prevailing stress conditions of the Mediterranean Basin (chiefly, high temperature during the growing season, low winter and spring temperatures, water shortage and nutrient deficiencies), where they can grow and produce fruit [3,6]. Owing to tolerance and adaptation of this crop to harsh environments, olive trees are nowadays planted in many areas of the world that are subject to high temperate and low precipitation regimes during the greater part of the growing season [7]. 

Olive fruits have a high K content, which is linked to a high tree K requirement [3]. Olive trees may develop dehydration symptoms in association with a low plant K status, chiefly in rain-fed plantations [3]. In addition, K deficiency in olive trees may inhibit stomatal closure, and compromise plant–water relations, especially under drought conditions [8]. 

Foliar nutrient sprays are widely applied to crop plants at different times of the growing season, principally to complement root fertilisation [9,10]. The mechanisms of foliar absorption are complex, and different leaf surface areas and structures may contribute differently to the uptake process, depending on factors such as species, stage of development or environmental factors at the time of treatment [11]. Nutrient solutions sprayed onto the foliage of plants may be absorbed via de cuticle and cuticular irregularities, through stomata, trichomes, veins and other epidermal structures [11]. 

Foliar K fertilisation may be a good strategy for supplying K to olive trees grown in drylands, where soil K availability is often limited [12]. Some field studies have evaluated the effect of several foliar K sprays supplied to olive trees at different times during the growing season, by assessing physiological, biochemical and olive quality factors [12,13,14,15,16,17]. Olive tree responses to foliar K treatments are often variable and limited, as also reported for K soil applications [18]. 

The penetration rates of different K salts have been evaluated with isolated astomatous pear and citrus leaf cuticles [19], excised trifoliate soybean leaves [20] and, more recently, with intact soybean plants and X-ray fluorescence microanalysis [21]. According to Schönherr [22], in foliar nutrition, anions and cations should penetrate in equivalent amounts, to maintain electrical neutrality. In such studies, the highest rates of foliar absorption of K salts were associated with compounds having a low point of deliquescence (POD), which is a phase transformation of a crystalline solid to a solution, taking place above a critical R.H. value [23]. By contrast, working with two model hygroscopic calcium (Ca) salts commonly used as foliar sprays, Fernández et al. [24] evaluated salt water sorption and desorption dynamics. Solid salt hydration was found to be affected by temperature, and the process of foliar spray drop drying was recognised to be related to the point of efflorescence (POE) of the compounds. This lower R.H. threshold (i.e., the POE) determines when a highly concentrated chemical solution will experience a phase transition into a solid state for crystallising [25].

Based on previous experiments with different crops, where we failed to gain evidence for foliar K absorption, and being aware of the importance of K for olives, we carried out a field trial, with the aim of assessing the rate of ion penetration of different K sources. The main scientific question we aimed to answer was whether foliar treatments with the most hygroscopic K salts would lead to the highest foliar K absorption rates. Our parting hypotheses were: (i) foliar treatment with the most deliquescent K salts (i.e., those with the lowest POE values) will lead to the highest olive leaf K concentrations; (ii) K sprays of compounds with different counter-ions will change olive leaf anion balance.

The proposed hypotheses are relevant because foliar nutrient spray absorption has been traditionally focussed on the uptake of essential elements which are generally cations [9]. If nutrient cation treatments also affect leaf anion balance, this may ultimately influence the process of epidermal cell wall transport. The cuticle and cell wall contain negative charges (mostly associated with hydroxyl and acid groups), and will act as a cation exchange membrane, and nutrient salt treatments may induce potential effects on cell membrane transporters, which may extrude ions to the surface. Hence, if verified, our hypotheses will contribute to improving our understanding of the foliar absorption process, and will help to optimize the efficacy of foliar treatments. 

## 2. Results

### 2.1. Physico-Chemical Properties of K Compounds

The properties of the K salts supplied as foliar sprays are shown in Table 1. The most soluble compound is K-carbonate, followed by K-chloride and K-nitrate, the least soluble compound being K-sulphate. All the anions were supplied at a 130 mM concentration; however, K-phosphate, K-sulphate and K-carbonate contained higher K concentrations (390 and 260 mM, respectively). Measured at a fixed temperature of 25 °C, the most hygroscopic compound is K-phosphate, which has a POE below 10% and a POD of 25%, followed by K-carbonate (45 and 35% POD and POE). By contrast, the least hygroscopic compounds are K-sulphate and K-nitrate, which have a POD ≥ 95% and a POE of 75 and 85%, respectively. The potential mechanisms of the drying of K spray droplets were evaluated from a theoretical viewpoint, by performing geochemical modelling with the PHREEQC code [26]. For carrying out calculations, a 1 mL volume of the K spray drops was preliminarily assumed. For achieving super-saturation of the K phases, the drops needed to lose more than 90% of their water volume, excepting K-sulphate, which would need to lose about 75% water. After reaching these water loss thresholds, the drop concentrations would be close to the described salt solubility concentrations (Table 1). This implies that at that point of water loss, the drop K concentration of the sprayed K compounds may vary from approximately 1.50 M (for K_2_SO_4_) to 22M (for K_2_CO_3_ and K_3_PO_4_), which are extremely high values.

### 2.2. Olive Leaf K and Anion Absorption

**Figure 1:**
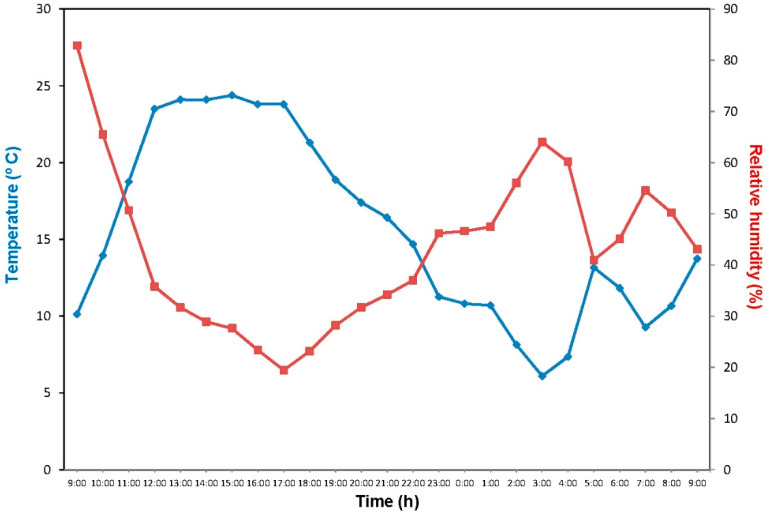
Air temperature (blue line) and R.H. (red line) values recorded at the olive tree plantation during the experimental period.

The potential absorption of K and its accompanying anions was evaluated after spraying shoots of Arbequina olive trees, by monitoring the R.H. and temperature conditions during the entire experimental period (Figure 1). During the time of the trial, the average air temperature was 15.5 °C, with a range between 6.1 °C (at 06:00 a.m.) to 24.4 °C (at 02:00 p.m.). Air R.H. ranged from 19.5 (at 05:00 p.m.) to 82% (at 09:00 a.m.), with an average of 43% (Figure 1).

From Figure 1, it can be derived that none of the sprayed drops of K-phosphate (POE < 10% at 25 °C) dried onto the leaf surfaces, and that the K-carbonate (POE = 35% at 25 °C) drops also remained liquid for most of the experimental period, excepting between 12 and 8 p.m. on the treatment day, when the R.H. was below 35%. The remaining spray drop deposits were subjected to wetting (i.e., when the R.H. was above the POD, which likely rose slightly with the lower air temperature recorded overnight) and drying cycles during the experimental period. 

None of the six K salts applied led to a significant increase in leaf K concentration, compared to the untreated leaves (Table 2). Similarly, the K and C concentrations in the leaves did not vary significantly between harvest times (3 and 24 h) for any of the treatments.

Anion concentrations sometimes increased or decreased in response to K salt application, while in other cases no differences between treatments were detected (Table 3). For example, the chloride concentration, measured 3 h after foliar K application, was significantly higher in plants sprayed with KCl compared to the rest of the treatments, indicating the uptake of this element by the foliage. On the other hand, leaf nitrate concentrations varied between treatments, and K-nitrate-sprayed leaves did not have the highest tissue nitrate amounts. Sulphate and phosphate concentrations were generally not modified by K spray application; however, 24 h after foliar K spraying, tissue concentrations decreased for sulphate and increased for phosphate, compared to leaves collected after 3 h (Table 3).

Regarding the concentration of K recovered in the leaf washing liquid, significantly higher concentrations were obtained in all foliar K treatments compared to untreated leaves, both 3 and 24 h after foliar application (Table 4).

Concerning the anions recovered in the washing liquid of leaves collected 3 and 24 h after foliar treatment, their concentration varied depending on the K salt supplied. Chloride concentration was higher in leaves treated with KCl compared to untreated control plants, but only in samples collected 3 h after foliar spraying. For the rest of the anions (i.e., NO_3_^−^, SO_4_^−2^, PO_4_^−2^), higher concentrations were detected in leaves treated with their corresponding salts (KNO_3_, K_2_SO_4_, K_3_PO_4_), both at 3 and 24 h after K treatment application. In general, there was a high concentration of chloride and sulphate in the washing liquid, regardless of the K treatment (Table 5). 

## 3. Discussion

In this study, we evaluated the foliar absorption rate of K salts with variable degrees of hygroscopicity, sprayed onto shoots of commercial Arbequina olive trees. This crop has a high K requirement [3], and the application of foliar K sprays is recommended, especially in rain-fed plantations [12]. Despite the high K concentration in some treatments (e.g., 390 mM K supplied as K_3_PO_4_), which during the drying process may reach values up to 23 M (for K_3_PO_4_) according to the geochemical modelling performed, no significant leaf K concentration increases could be detected. The tissue K concentrations provided in most foliar K application papers generally show the cumulative amount of K of leaves treated with several K sprays during the growing season, the leaves generally being collected and analysed long after foliar application [12,13,14,17]. The tissue K concentrations reported for olive leaves treated with several K sprays often led to moderate or no significant K increases. The K spray concentrations supplied in such investigations were sometimes higher than the ones we selected (e.g., up to 540 mM K [14]); however, the efficacy of K absorption by olive leaves seems to be low, especially considering the concentration process that takes place in the drops during water evaporation after foliar spraying, as theoretically estimated in our study. It must be noted that Restrepo-Díaz et al. [14] reported no significant effects of foliar K treatments supplied to K-sufficient olive plants, and that our trial was performed with well-fertilised, commercial olive trees, which might hence perform similarly to their hydroponically well-K-nourished plants.

Aware of the results we gained in preliminary trials developed with other crop plants, in which we were also not able to detect significant tissue K increments after foliar K application, we set the same anion concentration for comparing the efficacy of different K salts. Salt anions may play an important role in mineral element absorption, but this has been evaluated in few investigations, compared to cations [27]. According to Schönherr [22], anions and cations should cross plant surfaces in equivalent amounts, to maintain electrical neutrality. Among all the anions applied, we only gained evidence of foliar chloride absorption after KCl supply 3 h after treatment, but we noticed variations, depending on the applied K source, with special regard to nitrate (see Table 3). Recently, Corrêa et al. [21] evaluated the absorption of different foliar-applied K salts, using sensitive X-ray fluorescence microanalysis, and reported different absorption and transport rates in association with different counter-ions: higher rates of uptake were recorded for organic K-formate and K-acetate, compared to the lower values recovered after K-sulphate and K-nitrate application [21]. The penetration of different K compounds has also been evaluated with isolated leaf cuticles [19] and detached soybean leaves [20], but the results are difficult to interpret, because the materials analysed are not physiologically active anymore (e.g., K translocation from the site of application to other tissues may be limited), and may involve experimental artefacts in the case of cuticles [9]. 

Potassium plays a major role in balancing anion uptake, movement and accumulation in plants [1,28,29]. Apoplastic concentrations may range between 2 and 26 mM K [2], cytoplasmatic K values may be between 50 to 100 mM [30], and 300 to 400 mM K concentrations may be reached in guard cells when stomata are open [31]. It can be reckoned to what extent plants may enable the absorption of foliar-applied K, given its key physiological role. In addition, the supply of K to the foliage may alter the epidermal cell anion equilibrium, as derived from the tissue nitrate concentrations and the sulphate (both 3 and 24 h after treatment), and from the chloride (only 3 h after K salt application) concentrations recovered from the surfaces of leaves after leaf washing. Our results indicate that K supply with different counter-ions may trigger the extrusion of certain anions; thereby, the mechanisms associated with the absorption of foliar-applied nutrient salts may be more complex than expected, and may not be simply ruled by, e.g., maintaining electrical neutrality, as suggested by Schönherr [22]. For example, a major role of K in nitrate assimilation has been reported, and hence it is not surprising that we found variable nitrate concentrations in response to the different K salt treatments (Table 3). In addition, the differential absorption of the cation (K in our case) and the various anions supplied as salts, may change the physico-chemical variables of the drop solutions. For example, if K is not absorbed and chloride is taken up, the pH of the drop will rise. Our results suggest a potential role for epidermal membrane ion transporters, which should be investigated in future research. 

An additional variable, which has been associated with increased foliar uptake rates, is selecting salts with a low point of deliquescence (POD) [19,21]). Working at 25 °C, we here estimated the POD and, more importantly, the point of efflorescence (POE) of the different K salts that had been recently related to the process of foliar spray drop drying [24]. By monitoring the environmental conditions during the trial development, we concluded that chiefly the drops of sprayed K-phosphate, but also of K-carbonate, remained liquid on the surfaces of the leaves for the entire experimental period. Foliar absorption is meant to occur only when there is a liquid phase on top of the leaves [9]: hence, the occurrence of liquid drops of K carbonate and principally K-phosphate on the surfaces of the treated olive leaves, should have led to increased tissue K, phosphate and carbonate (measured and tissue C increments) concentrations, but this was not the case in our trial.

It is important to note that future work considering the POD and POE of compounds used as foliar fertilisers, should also focus on determining the kinetics of these transformations. Taking into account that KNO_3_ and K_2_SO_4_ have a close POE value (85 and 75%, respectively), when applying these two salts at the same T and R.H. conditions, it could be reckoned that KNO_3_ will crystallise earlier, because of its higher POE; however, considering that for crystallising, drops of the sprayed KNO_3_ solutions have to lose approximately 90% of their initial water volume compared to the c.a. 75% of K_2_SO_4_ drops, only sulphate could crystallise before nitrate: this will have to be analysed in future studies. Furthermore, this approach may also be helpful for predicting the performance of foliar-sprayed compounds such as K_2_CO_3_ (in our case), in situations when daily field R.H. fluctuations may remain for a long time close to the POE of the salt (as in our trial, Figure 1). Theoretically, spray drops may remain in liquid state during the whole experimental period, or become dry for a shorter time than expected. The POD values provided (measured at 25 °C) may significantly change with daily temperature fluctuations, and an estimation (especially in the range below 10 °C in our case) of the nutrient spray drop rewetting potential on the basis of the environmental variables recorded, should be interpreted with caution (e.g., for several chemicals, we observed that their POD was raised at lower temperatures). 

In conclusion, our results did not support our first hypothesis, that the most deliquescent K compounds would lead to the highest rates of foliar K absorption by olive leaves, since we determined no significant tissue K increases; however, our second proposed hypothesis, that K salt supply may change leaf ion balance, was verified in our trial. We again noticed that the rate of absorption of foliar-applied K could be difficult to detect by conventional methods, such as determining tissue K increments after foliar spraying, which may have been due to factors such as the high mobility plant of K or a limited rate of foliar K absorption. Physico-chemical variables such as the POE or POD of nutrient salts may play a role, but the scenario is so complex that it is currently difficult to ascertain which are the most important factor/s leading to improved foliar uptake rates and optimal plant responses. On the other hand, as shown in our study, leaves and epidermal cells may actively respond to exogenous cation and anion applications via ion absorption and/or extrusion, with the potential involvement of cell membrane transporters. It is hence concluded that more research is required, to characterise the mechanisms of foliar nutrient absorption and the main factors affecting foliar uptake mechanisms. 

## 4. Materials and Methods

### 4.1. Chemicals

The following potassium (K) salts were supplied as foliar sprays at a concentration of 130 mM: K-phosphate tribasic (K_3_PO_4_, reagent grade, ≥98%, Sigma–Aldrich, Germany); K-sulphate (K_2_SO_4_ ACS reagent, ≥99.0%, Sigma–Aldrich); K-nitrate (KNO_3_, ACS reagent, ≥99.0%, Sigma–Aldrich); K-chloride (KCl, ACS reagent, 99.0–100.5%, Sigma–Aldrich); K-carbonate (K_2_CO_3_, ACS reagent, ≥99.0%, Sigma–Aldrich); and K-bicarbonate (KHCO_3_, ACS reagent, 99.7%, Sigma–Aldrich).

The solubility (g kg^−1^) in water (at 25 °C) of all the compounds was taken from Haynes [32]. Estimations of the solubility (M) of the different chemicals (Table 1) and geochemical calculations were performed using the PHREEQC code and the Pitzer database [26].

All foliar treatments contained 0.1% Genapol X-80 (Sigma–Aldrich), which provided a mean surface tension of 27.21 ± 0.66 mN m^−1^ (*n* = 30). 

### 4.2. Point of Deliquescence (POD) and Efflorescence (POE) Estimation

The POD and POE of the different K salts was estimated using a climatic chamber with R.H. and temperature control (MKF 56, Binder, Germany). For finding the POE, 2 g of each solid salt was weighed and placed in a weighed Petri dish, which was kept open and located in the climatic chamber at a fixed temperature of 25 °C. The R.H. was gradually increased from 20 to 98% at different steps (5% every half an hour), checking if the salts were hydrated with an optical microscope. When approaching the POD, the R.H. was maintained for a longer period (1 h), for clearly observing that the salt was becoming liquid due to significant water sorption and dissolution. After having identified the POD of the salts, the R.H. was raised to 98%, and the samples were kept at this high humidity overnight. The process of water desorption and compound POE determination was carried out the next day, by gradually decreasing the R.H. from 98% R.H. at 5% step intervals, and checking the appearance of the solutions with the optical microscope until crystallisation was observed. Alternatively, the POE of non-hygroscopic compounds such as K-nitrate, was estimated by preparing concentrated solutions (close to their solubility value), and observing their crystallization as the R.H. was lowered from 98%, as described above. 

### 4.3. Plant Material

The absorption of foliar-applied K was evaluated at the end of September, in a commercial plantation (Viña Elena Jumilla, Murcia (38°25′5.5′′ N, 1°15′50.3′′ W)) of 8-year-old olive trees (*Olea europaea* L. var. Arbequina) grown within a frame of 3.5 × 1.5 m. The trees were grown on a calcareous, sandy-loam soil (approximately 70% sand, 15% silt and 15% clay), with 8.8 ± 0.02 pH, 0.38 ± 0.07% total nitrogen, 13.6 ± 0.39% calcium, 0.61 ± 0.04% K, 2.7 ± 0.01% magnesium, 0.07 ± 0.001% phosphorus and 0.16 ± 0.01% sulphur, as plant-available macronutrients. 

The prevailing R.H. and temperature conditions were monitored throughout the experimental period by a HOBO MX1101 sensor (Onset, Bourne, MA, USA). Before developing the trials, the plants were carefully selected and separated in groups of 3 trees per treatment. Three shoots of homogeneous size and healthy state were selected per tree. The experiment was organised following completely randomised block and foliar K treatments, which were only provided once, making sure that the leaves were well-wetted until observing spray run-off. Potassium sprays were supplied in the morning between 8 to 9 a.m., and leaf samples were collected after 3 and 24 h, respectively. 

### 4.4. Tissue Elemental Analysis

At the end of the experimental period, the olive shoots were collected, and the leaves from the different treatments were first washed in distilled water. This washing liquid was analysed by ICP-OES and liquid chromatography, as described below, for determining the leaf surface K and anion concentrations. 

For tissue elemental analysis, pre-washed olive leaves (i.e., after the preliminary water described above) were subsequently washed in a 0.1% detergent (Fairy, P&G), acidulated solution (0.1 N HCl). The plant tissues were then rinsed twice in tap water, and then in distilled water, and were consequently oven-dried at 70 °C for two days, then weighed and ground prior to mineral element determination after dry-ashing. The concentration of nutrients in the plant tissues was detected by inductively coupled plasma (ICP, PerkinElmer, Optima 3000), following the UNE-EN ISO/IEC 17025 standards for calibration and testing laboratories (CEBAS-CSIC Analysis Service, Murcia, Spain). On the other hand, the anions were analysed by liquid chromatography (CEBAS-CSIC Analysis Service, Murcia, Spain), after the extraction of approx. 0.3 g dry weight (D.W.) of leaf tissue in distilled water for 2 h, and centrifugation and filtration with 0.45 µm filters.

### 4.5. Data Analysis

Firstly, exploratory analyses of the data were carried out, to verify compliance with the assumptions of homoscedasticity, and the normality of the data for each situation was evaluated. To verify the homoscedasticity of the variances of the data, a Bartlett’s test was performed. In cases where the variances were not homogeneous, data transformations were performed. The normality of the data was checked with the Kolmogorov–Smirnov (KS) test. Differences in cation and anion concentrations in the leaves and washing liquids were analysed by performing two-way ANOVAs. Tukey HDS tests were carried out for estimating differences between factors when F-values were significant (*p* < 0.05). Statistical analyses were carried out using SPSS 15.0 (SPSS Inc., Chicago, IL, United States). 

## Figures and Tables

**Table 1:** Solubility, K and anion concentrations, approximate point of deliquescence (POD) and point of efflorescence (POE) at 25 °C of 130 mM K-phosphate, K-nitrate, K-sulphate, K-chloride, K-carbonate and K-bicarbonate supplied as foliar sprays.

Compound	Solubility (M)	[K] (mM)	[anion] (mM)	POD (%)	POE (%)
K_3_PO_4_	7.87	390	130	25	<10
KNO_3_	4.47	130	130	95	85
K_2_SO_4_	0.75	260	130	>95	75
KCl	5.59	130	130	85	70
K_2_CO_3_	10.94	260	130	45	35
KHCO_3_	4.25	130	130	85	65

**Table 2:** Potassium and C concentrations of olive leaves collected 3 and 24 h after 130 mM K-phosphate, K-nitrate, K-sulphate, K-chloride, K-carbonate and K-bicarbonate spraying. Data are means ± SD (*N* = 3; F (*p*) = Fisher test, with significance level in parentheses). In columns, different letters indicate significant differences (*p* < 0.05) according to Tukey’s HSD test.

Treatment	[K] (%)		[C] (%)	
	3 h	24 h	3 h	24 h
No K	0.88 ± 0.11 a	0.92 ± 0.08 a	47.66 ± 0.50 a	47.19 ± 0.35 a
K_3_PO_4_	1.00 ± 0.22 a	1.11 ± 0.10 a	47.90 ± 0.34 a	48.11 ± 0.51 a
KNO_3_	1.10 ± 0.26 a	0.98 ± 0.08 a	47.54 ± 0.30 a	47.25 ± 0.14 a
K_2_SO_4_	0.96 ± 0.16 a	1.03 ± 0.15 a	47.42 ± 0.53 a	47.54 ± 0.27 a
KCl	1.05 ± 0.19 a	1.13 ± 0.01 a	47.35 ± 0.52 a	47.54 ± 0.14 a
K_2_CO_3_	1.01 ± 0.13 a	1.04 ± 0.19 a	47.45 ± 0.51 a	47.80 ± 0.35 a
KHCO_3_	1.03 ± 0.65 a	1.12 ± 0.26 a	47.35 ± 0.52 a	47.81 ± 0.63 a
Treatment (T)	0.96 (0.468)		1.64 (0.172)	
Hours (H)	0.77 (0.388)		0.44 (0.514)	
T × H	0.32 (0.921)		1.05 (0.417)	

**Table 3:** Anion concentrations of olive leaves collected 3 and 24 h after 130 mM K-phosphate, K-nitrate, K-sulphate, K-chloride, K-carbonate and K-bicarbonate spraying. Data are means ± SD (*N* = 3; F (*p*) = Fisher test, with significance level in parentheses). In the columns, the different letters indicate significant differences (*p* < 0.05) according to Tukey’s HSD test.

Treatment	[Cl^−^] (mg g^−1^)		[NO_3_^−^] (mg g^−1^)		[SO_4_^2−^] (mg g^−1^)		[PO_4_^3−^] (mg g^−1^)	
	3 h	24 h	3 h	24 h	3 h	24 h	3 h	24 h
No K	0.75 ± 0.12 a	0.65 ± 0.07 a	0.15 ± 0.03 b	0.13 ± 0.01 b,c	1.85 ± 0.24 a	1.64 ± 0.49 a	3.46 ± 0.64 a	5.44 ± 0.71 a
K_3_PO_4_	0.92 ± 0.05 a	0.84 ± 0.13 a	<0.02	<0.02	1.22 ± 0.25 a	1.01 ± 0.03 a	3.77 ± 0.16 a	5.12 ± 2.47 a
KNO_3_	0.87 ± 0.18 a	0.69 ± 0.05 a	0.13 ± 0.02 b	0.10 ± 0.05 b	1.43 ± 0.70 a	1.22 ± 0.44 a	2.83 ± 0.15 a	4.67 ± 1.71 a
K_2_SO_4_	0.92 ± 0.27 a	0.68 ± 0.03 a	0.07 ± 0.02 a	0.10 ± 0.02 b	1.37 ± 0.43 a	1.12 ± 0.45 a	3.47 ± 0.90 a	5.64 ± 1.57 a
KCl	1.35 ± 0.17 b	0.83 ± 0.13 a	0.14 ± 0.03 b	0.16 ± 0.03 c	1.63 ± 0.24 a	1.28 ± 0.12 a	3.64 ± 0.67 a	4.96 ± 0.50 a
K_2_CO_3_	0.64 ± 0.07 a	0.77 ± 0.10 a	0.06 ± 0.03 a	0.04 ± 0.01 a	1.36 ± 0.19 a	1.28 ± 0.24 a	2.56 ± 1.06 a	4.88 ± 0.96 a
KHCO_3_	0.75 ± 0.03 a	0.78 ± 0.12 a	0.10 ± 0.01 a	0.17 ± 0.01 c	1.95 ± 0.36 a	1.36 ± 0.20 a	3.65 ± 0.76 a	5.48 ± 1.53 a
Treatment (T)	6.79 (<0.001)		11 (<0.001)		3.3 (0.014)		0.59 (0.734)	
Hours (H)	12.17 (0.002)		0.92 (0.349)		4.42 (0.045)		25.99 (<0.001)	
T × H	3.99 (0.005)		3.1 (0.029)		0.45 (0.839)		0.16 (0.985)	

**Table 4:** Potassium concentrations recovered in the washing liquid of olive leaves treated with 130 mM K-phosphate, K-nitrate, K-sulphate, K-chloride, K-carbonate, and K-bicarbonate foliar sprays. Samples were collected 3 and 24 h after foliar spraying. Data are means ± SD (*N* = 3; F (*p*) = Fisher test, with significance level in parentheses). In the columns, the different letters indicate significant differences (*p* < 0.05) according to Tukey’s HSD test.

Treatment	[K] (mg L^−1^ m^−2^)	
	3 h	24 h
No K	6.44 ± 2.58 a	12.86 ± 4.01 a
K_3_PO_4_	287.75 ± 28.76 e	324.69 ± 35.76 d
KNO_3_	91.72 ± 10.68 b	100.23 ± 8.03 b
K_2_SO_4_	204.24 ± 12.25 d	197.63 ± 24.62 c
KCl	66.39 ± 7.3 b	74.61 ±123.41 b
K_2_CO_3_	138.61 ± 9.7 c	194.03 ± 19.68 c
KHCO_3_	80.97 ± 15.09 b	94.18 ± 16.01 b
Treatment (T)	170 (<0.001)	
Hours (H)	9.14 (0.005)	
T × H	1.96 (0.105)	

**Table 5:** Chloride, nitrate, sulphate and phosphate ion concentrations recovered after washing olive leaves treated with 130 mM K-phosphate, K-nitrate, K-sulphate, K-chloride, K-carbonate or K-bicarbonate. Samples were collected 3 and 24 h after foliar spraying. Data are means ± SD (*N* = 3; F (*p*) = Fisher test, with significance level in parentheses). In columns, different letters indicate significant differences (*p* < 0.05) according to Tukey’s HSD test.

Treatment	[Cl^−^] (mg L^−1^ m^−2^)		[NO_3_^−^] (mg L^−1^ m^−2^)		[SO_4_^2−^] (mg L^−1^ m^−2^)		[PO4^−3^] (mg^−1^ m^−2^)	
	3 h	24 h	3 h	24 h	3 h	24 h	3 h	24 h
No K	91.9 ± 42.2 a	300.7 ± 107.9 a	10.4 ± 4.4 a	18.6 ± 6.4 a	142.1 ± 37.4 a	266.2 ± 96.7 a.b	5.3 ± 2.4 a	7.1 ± 3.2 a
K_3_PO_4_	198.6 ± 13.9 a,b	179.5 ± 73.9 a	8.7 ± 2.2 a	6.2 ± 5.6 a	157.8 ± 11.5 a	143.8± 60.6 a	268.7 ± 41.2 b	280.0 ± 38.0 b
KNO_3_	283.1 ± 19.8 a,b	229.5 ± 80.4 a	148.5 ± 3.8 b	166.3 ± 21.9 b	269.2 ± 17.0 a,b	218.1 ± 44.9 a,b	3.04 ± 3.5 a	1.0 ± 0.0 a
K_2_SO_4_	165.5 ± 11.8 a,b	197.8 ± 39.7 a	12.0 ± 1.9 a	11.7 ± 1.5 a	390.6 ± 46.7 b	387,2± 28.3 b	1.0 ± 0.0 a	1.0 ± 0.0 a
KCl	346.5 ± 88.1 b	337.4 ± 33.5 a	13.2 ± 2.9 a	16.6 ± 2.4 a	240.8 ± 66.3 a,b	250.9 ± 32.0 a,b	1.0 ± 0.0 a	5.2 ± 3.7 a
K_2_CO_3_	153.2 ± 13.7 a,b	295.7 ± 82.2 a	8.7 ± 1.7 a	15.1 ± 3.2 a	136.2 ± 12.8 a	258.8 ± 4.2 a,b	7.0 ± 1.7 a	7.6 ± 1.5 a
KHCO_3_	180.9 ± 36.7 a,b	130.0 ± 36.1 a	10.7 ± 2.5 a	10.3 ± 2.8 a	153.3 ± 30.3 a	110.8 ± 28.2 a	1.0 ± 0.0 a	1.0 ± 0.0 a
Treatment (T)	3.35 (0.013)		399 (<0.001)		8.25 (<0.001)		277 (<0.001)	
Hours (H)	2.38 (0.134)		5.02 (0.033)		2.3 (0.138)		0.28 (0.599)	
T × H	1.86 (0.124)		1.6 (0.184)		2 (0.099)		0.13 (0.992)	

## Data Availability

All data are included in the manuscript, and are available upon request to the authors.
